# Rural retention of doctors graduating from the rural medical education project to increase rural doctors in Thailand: a cohort study

**DOI:** 10.1186/s12960-015-0001-y

**Published:** 2015-03-01

**Authors:** Nonglak Pagaiya, Lalitaya Kongkam, Sanya Sriratana

**Affiliations:** Sirindhorn College of Public Health, Khon Kaen, 90/1 Anamai Road, Muang, Khon Kaen, 40000 Thailand; National Health Security Office, Muang, Nakhon Ratchasima, 30000 Thailand; International Health Policy Program, Ministry of Public Health, Tiwanond Road, Muang, Nonthaburi, 11000 Thailand

**Keywords:** Human resources for health, Doctors, Rural retention, Survival analysis

## Abstract

**Background:**

In Thailand, the inequitable distribution of doctors between rural and urban areas has a major impact on access to care for those living in rural communities. The rural medical education programme ‘Collaborative Project to Increase Rural Doctors (CPIRD)’ was implemented in 1994 with the aim of attracting and retaining rural doctors. This study examined the impact of CPIRD in relation to doctor retention in rural areas and public health service.

**Methods:**

Baseline data consisting of age, sex and date of entry to the Ministry of Health (MoH) service was collected from 7,157 doctors graduating between 2000 and 2007. There were 1,093 graduates from the CPIRD track and 6,064 that graduated through normal channels. Follow-up data, consisting of workplace, number of years spent in rural districts and years within the MoH service, were retrieved from June 2000 to July 2011. The Kaplan-Meier method of survival analysis and Cox proportional hazards ratios were used to interpret the data.

**Results:**

Female subjects slightly outnumbered their male counterparts. Almost half of the normal track (48%) and 33% of the CPIRD doctors eventually left the MoH. The retention rate at rural hospitals was 29% for the CPIRD doctors compared to 18% for those from the normal track. Survival curves indicated a dramatic drop rate after 3 years in service for both groups, but normal track individuals decreased at a faster rate. Multivariate Cox proportional hazards modelling revealed that the normal track doctors had a significantly higher risk of leaving rural areas at about 1.3 times the CPIRD doctors. The predicted median survival time in rural hospitals was 4.2 years for the CPIRD group and 3.4 years for the normal track. The normal track doctors had a significantly higher risk of leaving public service at about 1.5 times the CPIRD doctors.

**Conclusions:**

The project evaluation results showed a positive impact in that CPIRD doctors were more likely to stay longer in rural areas and in public service than their counterparts. However, turnover has been increasing in recent years for both groups. There is a need for the MoH to review and improve upon the project implementation.

## Introduction

The effective mobilisation of the health workforce is essential in improving the performance of the health system and achieving key health objectives, particularly in low- and middle-income countries [1]. The World Health Organization (WHO) stated that there were approximately 57 countries with critical shortages of doctors, nurses and midwives, particularly in sub-Saharan Africa and Southeast Asia [2]. Thailand is no exception.

Thailand is divided geographically into 76 provinces and the capital city of Bangkok. For administrative purposes, each province is divided into townships, districts, sub-districts and villages. Districts, sub-districts and villages are classified as rural areas. The health service system in Thailand is publicly dominated, where public hospitals account for 78% of all hospitals (1,142 out of 1,464) and hospitals serving under the Ministry of Health (MoH) account for approximately 67% of all public and private hospitals [3]. To supplement the public sector, private hospitals play significant roles in providing health services in urban areas. Community hospitals are health facilities located at the district level providing secondary health services and categorised as rural health facilities. All other hospitals are located in urban areas. In the total of 878 rural districts, there are 742 community hospitals (84%), and all of them are managed by the MoH. Therefore, community hospitals are defined as facilities in rural areas, and hospitals under the MoH are defined as public sector facilities.

The persistent maldistribution of doctors continues to have a major impact on access to care for those living in the rural, remote or underserved communities of Thailand. This can be seen from the fact that doctor density (defined as the number of doctors per 1,000 population) in Bangkok in 2007 was 10 times higher than that in the northeast, the most rural region of Thailand [3]. The population in rural areas has limited access to doctors; only 16.5% of the doctors work in rural areas where 54% of the population lives [4]. However, the number of doctors graduating has gradually increased, from 1,250 in 2000 to 1,540 in 2007 [3]. Of these, approximately 75% of the new graduates entered the MoH service annually [3].

Over the past four decades, the Government of Thailand has implemented several strategies to attract and retain doctors in rural areas [5]. These include coercion measures, financial and non-financial measures and educational interventions. Historically, a low proportion of medical graduates have worked in rural hospitals after gaining qualifications. In an attempt to improve rural health services, the government introduced a minimum period of 3 years of compulsory public service in 1971. This mandated that new graduates must work in the MoH public service, particularly in rural hospitals, for the first 3 years of their careers.

A financial strategy was also implemented in 1975 and continued to supplement the income of doctors posted to rural areas with a hardship allowance, non-private practice allowance and professional allowance. In 2008, the total monthly income for newly graduated doctors working in rural hospitals was US$ 1,900 per month, 10% to 15% higher than that for new doctors working in urban areas [6]. Total income varied according to geographic remoteness and the length of rural service time. The monthly income of rural doctors was higher than that of other professionals working in the rural hospitals, but still lower than that of doctors working in the private sector.

Non-financial incentives were also implemented. Career advancement was offered to senior doctors who had served in rural districts for a long period. From 1970, specialist training was made available to doctors working in rural areas. Other non-financial measures implemented included health infrastructure development, medical supply and equipment provisions and the establishment of referral and consultation systems [5].

Educational interventions were among the prior approaches used by the government, especially to increase the number of doctors graduating. Medical student intake increased from 1,528 in 1997 to 2,282 per year in 2013, to satisfy the rising demand for doctors [3]. An important educational initiative was introduced in 1974, which favoured the recruitment of medical students from rural areas. This grew to fruition in 1994 as the ‘Collaborative Project to Increase Rural Doctors (CPIRD)’. The project’s aim was to increase the number of rural doctors by increasing medical education opportunities for students with a rural background. In addition, to train doctors in line with the health system needs as well as to improve staffing capacity at MoH hospitals, the MoH collaborated with medical schools in developing and implementing the project. Each year, approximately 300 students were recruited into medical schools with emphasis on rural backgrounds and academic proficiency [7]. However, because of limitations in recruiting qualified students from rural areas, the medical student intake was less than expected in the early years. The students were trained at medical schools and MoH hospitals close to their hometowns. They were then obliged to return to their home provinces upon graduation. The 6-year curriculum was split into two parts: students spent the first 3 years studying pre-clinical science subjects at the medical schools and then the final 3 years at regional or general hospitals within the MoH. Once graduated, CPIRD doctors were posted back to their home provinces. Normal track graduates were then allowed to choose from the remaining available positions. Together with the graduates on normal track, the CPIRD graduates were required to work within the MoH, particularly at rural hospitals, for 3 years after graduation. A monetary fine, equivalent to US$ 12,500, was applied to both sets of graduates in the event of breach of contract.

Rural medical education programmes to address the shortage of rural doctors have proved significant in the recruitment and retention of doctors in the rural areas of developed countries [8-10]. A rural medical education programme has been defined as a medical training programme aiming to increase the likelihood of retaining their services in rural and remote areas once qualified [9]. The programmes focus on student selection, based on rural background criteria. Some programmes combine rural selection with rural-orientated curricula (strategies to stimulate interest and participation in community-based medicine, including clinical rotation in a rural setting) and/or hometown placement after graduation [9]. The majority of these studies were conducted in developed countries, some were in developing countries, but most of these had methodological limitations. Previous research focused mainly on job satisfaction or intention to leave rural practice, rather than on actual observed behaviour. Little was known about the success of the rural medical education programme and its impact on rural retention against normal track doctors. Therefore, a rigorous study was required to assess the impact of the rural medical education programme. This study was carried out to assess two main areas of interest: 1) the effect of the CPIRD on rural retention compared with the normal stream of training for public-sector-employed graduates and 2) the CPIRD influence on public sector retention compared with the normal stream. The results generated by survival analysis of the health workforce data will benefit future rural health workforce planning and assist in the development of improved retention strategies.

## Methods

The study population was limited to doctors who entered the MoH after graduation. This cohort study used baseline data of age, sex and year of joining the MoH for 7,157 graduates between the years 2000 and 2007. Doctors who graduated under CPIRD were labelled as ‘CPIRD’. Those not under CPIRD were labelled as ‘normal track’ or ‘non-CPIRD’. There were 1,093 doctors that graduated from the CPIRD track and 6,064 that graduated from the normal track. Follow-up data consisted of workplace, number of years spent in rural practice, the year of exit from rural hospitals and the year of exit from MoH service or public service. The data were collected between June 2000 and July 2011. Individual data was originally obtained from the administrative data in the MoH information system. All medical doctors practising in the MoH are obliged under civil service regulations to record their career details in this database, and it is updated annually. As of July 2011, all doctors in the study had worked for more than 3 years. To evaluate the longitudinal change with time in the rural/urban proportions and the public service retention of the doctors, information on workplace during 2000 and 2011 was used. Follow-up rates were 100% for the entire study population.

This study defined rural areas as practice in community hospitals located in rural districts. Rural districts are classified by geographical distance from a city or town, low population density and low revenue generation. There are 878 rural districts with 742 community hospitals throughout Thailand [3]. All other health facilities were considered as being in urban areas. Doctors were labelled as ‘rural retention’ if their current workplaces, as of July 2011, were at community hospitals. Those whose place of work, as of July 2011, was at any hospital under the MoH were labelled as ‘public retention’. Data analysis took place in July 2011, following the annual database update from March to June 2011.

Survival analysis methods were considered as effective tools to measure doctor turnover and retention in rural areas [11]. Survival analysis measures the time until an event occurs. In this study, the event of interest was the time involved between taking up an MoH position to the time of leaving a community or MoH hospital. A ‘failure or not-retained’ event was defined as a doctor leaving a community hospital or MoH hospital, while a ‘censored or retained’ event was defined as a doctor remaining in a rural health facility or MoH hospital at the end of the study observation period. A ‘not-retained’ under this definition would be a doctor going for specialty training under urban facility quotas; however, a doctor going for specialty training under a rural facility scheme was considered as ‘retained’. For the purposes of public service retention, going for specialty training was defined as ‘retained’.

The Kaplan-Meier method of survival analysis was used to analyse the data. This technique enables employment data for all doctors who have worked in rural health facilities or public service during the period of interest to be included in the analysis. Main outcome measurements were Cox proportional hazards ratios, comparative risk of CPIRD doctors leaving rural health facilities and the public service compared to normal track doctors and predicted median survival (the predicted time in years from the commencement of appointment until half the workforce had left). A Stata package was used for data input and analysis.

Ethics approval was received from the Khon Kaen Sirindhorn College of Public Health Human Research Ethics Committee.

## Results

The study population was comprised of 7,157 doctors who graduated and joined the MoH service between 2000 and 2007. Of these, 1,093 graduated from the CPIRD track and 6,064 from the normal track. Overall, female doctors slightly outnumbered male doctors, as shown in Table 1. Almost half of the doctors (45.9%) left the MoH service, 33% of CPIRD and 48% of normal track, respectively. Of all doctors, 34% were still working at general or regional hospitals located in urban areas, with 20% at rural hospitals. The retention at rural hospitals was 29% for CPIRD doctors compared to only 18% for normal track doctors. Table 1 also shows the number of doctors entering the MoH service by year. From 2000 to 2002, there were fewer CPIRD doctor graduates, due to limited student intake; the number of graduating doctors has increased each year since then. There were approximately 200 new CPIRD doctors each year. The number of normal track doctors entering the MoH service gradually increased, ranging from 438 in 2000 to 884 in 2007.

**Table 1:** **Doctor characteristics by CPIRD and normal track**

**Characteristics**	**Total**	**CPIRD track**	**Normal track**
Sex			
- Male	3,378 (47.2)	457 (41.8)	2,921 (48.2)
- Female	3,779 (52.8)	636 (58.2)	3,143 (51.8)
Current workplace			
- Community hospitals	1,430 (20.0)	316 (28.9)	1,114 (18.4)
- General/regional hospitals	2,442 (34.1)	420 (38.4)	2,022 (33.3)
- Left MoH	3,285 (45.9)	357 (32.7)	2,928 (48.3)
Year of MoH entry			
- 2000	444	6	438
- 2001	600	10	590
- 2002	755	32	723
- 2003	925	129	796
- 2004	992	143	849
- 2005	1,131	249	882
- 2006	1,163	261	902
- 2007	1,147	263	884

### Rural retention

In the analysis of rural retention, 522 data sets of individual doctors were excluded as data for the year exiting from a rural hospital or the MoH service were not available. These pertained to 49 CPIRD track and 473 normal track doctors. From the CPIRD, 7 doctors left the MoH service and 42 currently work in general or regional hospitals. From the normal track, 67 resigned from the MoH service and 406 currently work in general or regional hospitals.

Throughout the 11-year period (June 2000 to July 2011), 28,177.6 doctor-years of observation time were analysed. The incidence rate (IR) of doctors who left rural areas was 17.6% of doctor-years, with an overall median survival time (the length of time in years until half the doctors had left the rural areas) of 3.7. CPIRD and normal track doctors had observation times of 4,316.3 and 23,861.3 doctor-years, respectively. The incidence rate of doctors who left the rural areas was lower for CPIRD than for normal track at 14.9% (95% CI = 13.7, 16.1) and 18.2% (95% CI = 17.6, 18.7) doctor-years, respectively. The incidence rate (per 100 doctor-years) increased in both groups: the CPIRD track from 2.2% for the 2001 cohort to 17.0% for the 2007 cohort and the normal track from 8.1% for the 2000 cohort to 28.3% for the 2007 cohort.

The predicted median survival (the length of time until half the workforce had left) revealed that the overall length of stay in rural areas was 4.2 years for CPIRD track doctors and 3.4 years for normal track doctors. However, median survival time decreased in the later cohorts. CPIRD track doctors had a median survival time of 8.0 years for the 2002 cohort which decreased to 3.9 years for the 2007 cohort. Normal track doctors had a high median survival time of 8.4 years for the 2000 cohort, but this dropped to 3.1 for the 2007 cohort. Results are shown in Table 2.

**Table 2:** **Median survival time (years) in rural areas of CPIRD and normal track doctors by year of entering the MoH service (between June 2000 and July 2011)**

**Entering MoH**	**CPIRD track**			**Normal track**		
	**Doctor-years**	**IR/100**	**Median time (95% CI)**	**Doctor-years**	**IR/100**	**Median time (95% CI)**
2000	44.6	—	—	3,203.8	8.1	8.4 (7.8, 8.9)
2001	89.1	2.2	—	3,301.1	11.6	6.4 (6.1, 6.8)
2002	199.0	7.5	8.0 (6.3, 9.7)	3,564.6	13.6	5.0 (4.7, 5.4)
2003	562.3	13.3	5.0 (4.7, 5.3)	3,168.7	18.8	3.3 (3.1, 3.6)
2004	632.5	14.7	4.0 (3.6, 4.4)	3,064.5	21.7	3.2 (3.1, 3.2)
2005	1,056.9	14.8	4.8 (4.4, 5.3)	2,827.3	24.4	3.1 (3.0, 3.1)
2006	903.8	17.7	4.0 (3.7, 4.3)	2,608.4	25.2	3.1 (3.1, 3.1)
2007	828.1	17.0	3.9 (3.6, 4.2)	2,123.0	28.3	3.1 (3.0, 3.1)
Total	4,316.3	14.9	4.2 (3.9, 4.4)	23,861.3	18.2	3.4 (3.3, 3.5)

**Figure 1:**
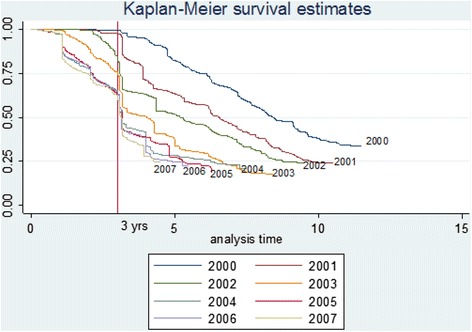
**Doctor survival curves of rural area retention by year of entering the MoH service (between June 2000 and July 2011).**

Survival curves shown in Figure 1 indicate that all cohorts dropped dramatically after 3 years in service. Later cohorts had a tendency to leave the rural areas earlier than their predecessors. Numbers from the first two intakes (2000 and 2001) dropped greatly after 3 years in service, and rural survival curves gradually declined to 25% during the 11-year study period. The 2002 cohort started to leave the rural areas after only 2 years in service, and this increased dramatically after 3 years. The 2003 to 2007 groups began to leave after only 1 year and this also increased after 3 years, with less than half remaining in the rural areas for a fourth year of MoH service.

**Figure 2:**
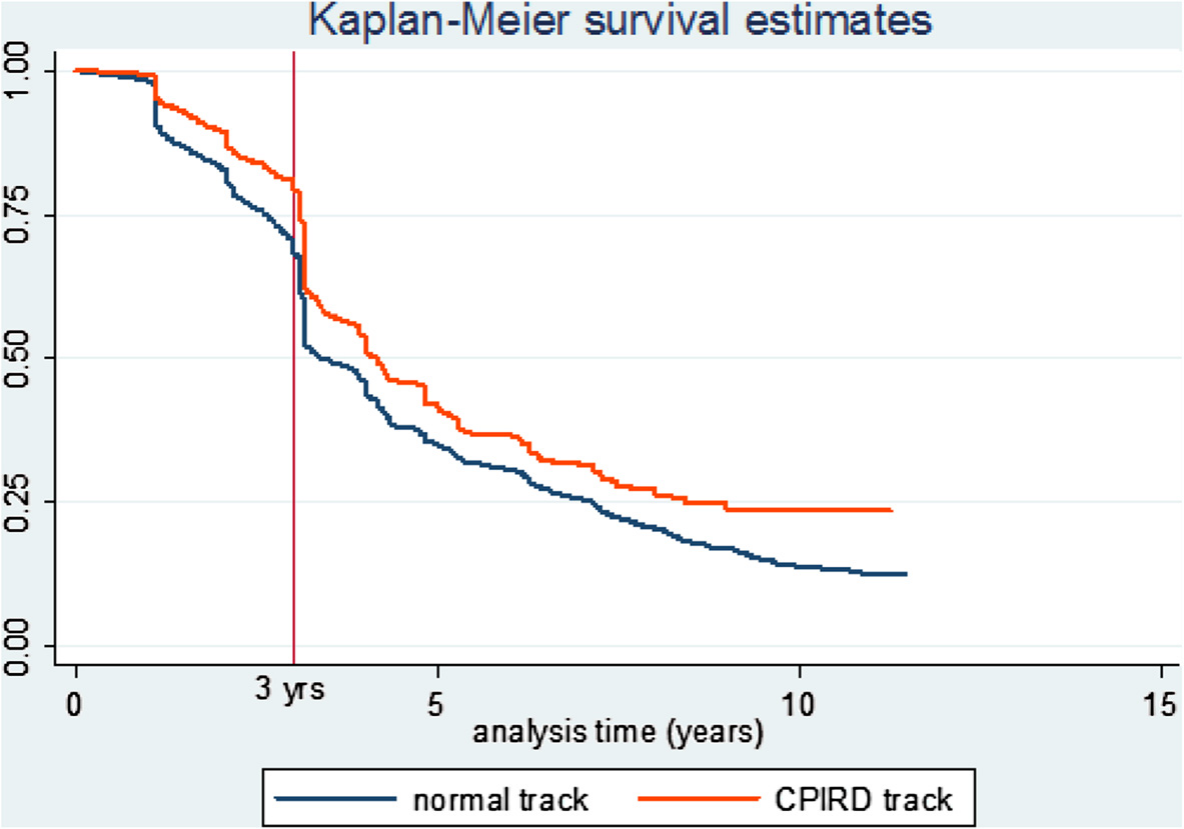
**Survival curves of rural area retention by CPIRD and normal track doctors (between June 2000 and July 2011).**

Survival curves of rural retention for both CPIRD and normal track doctors are shown in Figure 2. These indicate that during the 11-year study period (June 2000 to July 2011), approximately 24% and 13% of CPIRD and normal track doctors remained in rural areas, respectively. Survival curves of both tracks gradually declined after 1 year with 80% of CPIRD and 69% of normal track doctors remaining in rural areas at the end of the third year. Subsequently, both tracks significantly declined, and by the fourth year, only 51% of CPIRD and 44% of normal track doctors remained in the rural areas. Multivariate Cox proportional hazards modelling revealed that CPIRD doctors had a significantly increased risk of leaving the rural areas of 0.795 times that of normal track doctors, or conversely, the normal track doctors had a significant risk of leaving rural areas of 1.3 times higher than the CPIRD track doctors. Results are shown in Table 3.

**Table 3:** **Predictors of doctor retention in rural areas and public service resulting from survival analysis (Cox regression)**

**Retention**	**Hazard ratio**	**SE**	***P*** **value**	**95% CI**
Rural retention				
- Normal track	1	—	—	—
- CPIRD track	0.795	0.034	<0.001	0.73, 0.86
Public service retention				
- Normal track	1	—	—	—
- CPIRD track	0.665	0.036	<0.001	0.67, 0.74

### Public service retention

**Figure 3:**
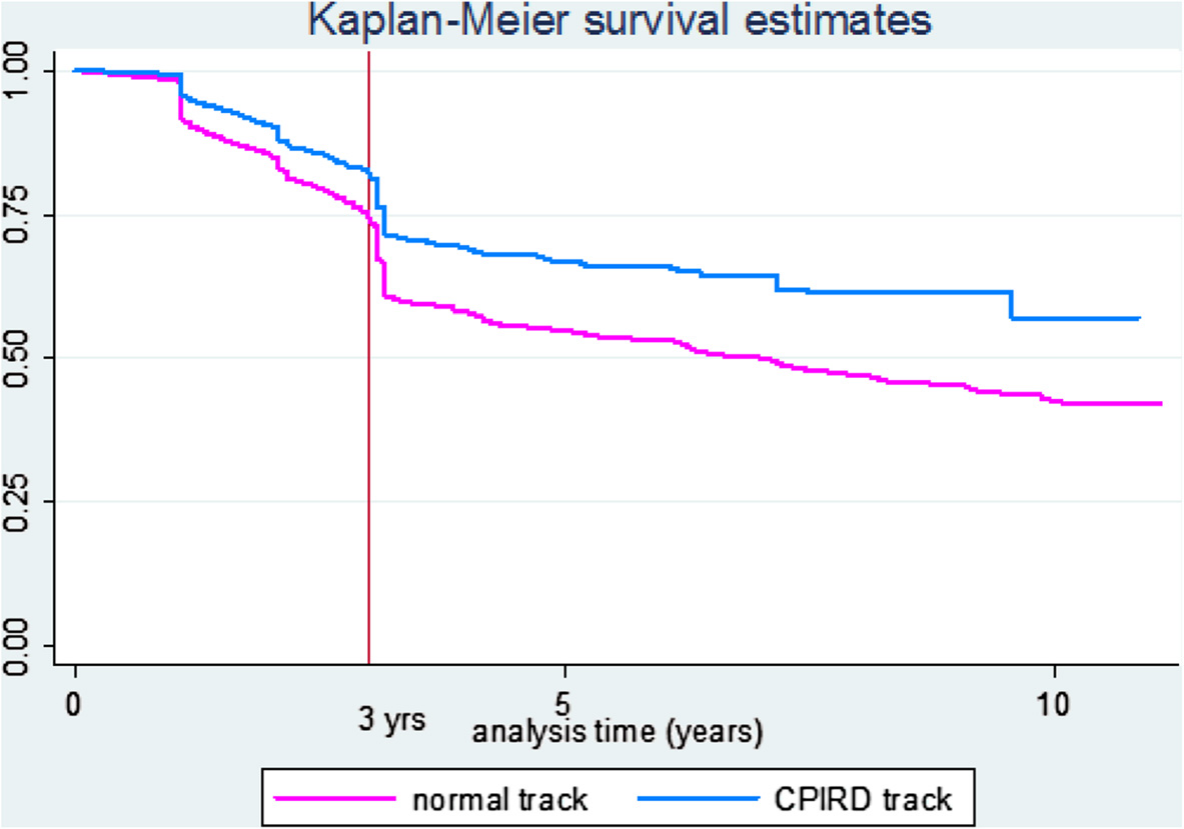
**Survival curves of MoH service retention by CPIRD and normal track doctors (between June 2000 and July 2011).**

Survival curves of public service retention are shown in Figure 3. Results are similar to the rural retention and show a significant decline over the 11-year period. After 1 year, both tracks gradually left the public service. Approximately 74% of normal track and 82% of CPIRD track doctors remained in public service for a third year. Subsequently, both tracks dropped dramatically until, by the 10th year, 43% of normal and 57% of CPIRD track doctors were likely to be still employed in public service.

The multivariate Cox proportional hazards ratio for public service retention was 0.665. This indicated that the normal track doctors had a significantly increased risk of leaving the public service at 1.5 times more than the CPIRD track doctors. Results are shown in Table 3.

## Discussion

The study was limited by the nature of the administrative database, which was not created for research purposes. The availability of the individual characteristics of the doctors that might affect both public and rural retention was also limited, i.e. hometown of origin, socio-economic background. This analysis therefore failed to recognise individual characteristic factors. The majority of doctors (75%) enter the MoH service immediately after graduation; however, the study excluded those who initially worked for other agencies. Therefore, this study did not accurately represent all the doctors.

The study assessed actual observations and carried out rigorous analysis to evaluate doctor retention trends. The CPIRD approach combined three strategies to attract and retain doctors in rural areas: selective admission criteria focusing on a rural background, collaborative training between medical schools and the MoH, and preferential job placement in their home provinces after graduating. This study showed that the CPIRD programme increased the retention time for rural doctors from 3.4 to an average of 4.2 years. The average rural retention time of normal track doctors mirrored the results of Russell et al. [12], who found that the median rural retention time of doctors was about 3 years. Though CPIRD track doctors remained in the rural areas and public service longer than the normal track, both groups gradually left the rural areas after only 1 year in service. The length of time until half the doctors had left rural areas for the CPIRD group was longer than for the normal track. However, the rural retention of CPIRD and normal track doctors became comparable with increasing time. Some doctors moved away from the rural areas before the 3-year compulsory period. The main pull factor was to further their specialty training [13]. A potential explanation was that, over the study period, medical schools significantly increased the specialist-training quota; this approach could attract a young doctor cohort to leave rural practice earlier.

One possible reason that resulted in normal track doctors leaving rural areas earlier than their CPIRD counterparts might be because CPIRD doctors were favoured over the normal track in choosing their workplaces. CPIRD doctors were assigned back to their home provinces preferentially, before the normal track doctors could choose from the remaining workplaces available. This has sent some alarming signals to the government and those who were responsible for the CPIRD project to improve project implementation. Another possible way to improve rural retention would be to increase the specialist positions available at rural hospitals. Then specialist doctors could have the option to return to rural areas after graduation. Increasing rural area exposure during medical training could also benefit and assist in preparing doctors for work in the rural areas.

Significant differences in the risk of leaving between CPIRD and normal track doctors were not surprising, as similar results were found by studies conducted in developed countries. A critical review by Wilson et al. [9] indicated that, of all the criteria used to attract and retain doctors in rural and remote areas, a well-defined selection (selection criteria evaluation including geographic origin and rural background) and education policy to optimise the medical training programmes will have the most positive impact. In addition to this critical review, other studies have confirmed that recruiting medical students from rural communities increases the likelihood that they will choose to work in a rural practice [10,14], so the results from this study are not exceptional.

Positive educational strategies increase the number of doctors in rural practice. A systematic review of six well-established rural track training programmes, aimed at increasing the supply of rural doctors in the United States of America, demonstrated that the number of graduates practising in rural areas ranged from 53% to 64% [8]. The CPIRD project is a Thai targeted educational approach to attract and retain doctors in rural areas. The project has increased the number of doctors in rural practice, and the results have agreed with the systematic review [8] and reiterated those of other studies [10,15].

In relation to public service retention, 3 years of public service were obligatory for both CPIRD and normal track doctors. The 11 years of longitudinal data revealed doctor mobility trends. Most of the graduates completed the 3-year compulsory public service requirement. This supports the systematic review by Wilson et al. [9] that coercive strategies can address short-term recruitment needs. However, retention dropped dramatically once the 3-year compulsory period ended. This indicated that compulsory public service alone did not have an impact on long-term public retention. However, over the 11-year observation, public retention of CPIRD doctors was higher than that of normal track doctors and analysis showed that the normal track doctors had a significant increased risk of leaving public service compared to the CPIRD doctors. These results supported and recommended the effectiveness of the combination between rural track doctors and coercive strategies.

## Conclusions

The Thai CPIRD project combined three strategies: a rural admissions process, collaborative training between medical schools and the MoH, and preferential return to service in their home provinces once graduated. The compulsory public service measure retained doctors in the public sector and rural health facilities in the short term. However, retention time increased when combined with rural track doctor education approaches. Though the project evaluation results showed that CPIRD doctors were likely to remain longer in rural hospitals, overall rural turnover is still high. This has sent some signals to the MoH that they must review and improve upon project implementations.

## Abbreviations

CPIRD: Collaborative Project to Increase Rural Doctors
IR: Incidence rate
MoH: Ministry of Health
